# Supplementary material for: Unveiling the Bioactive Potential of the Invasive Jellyfish Phyllorhiza punctata Through Integrative Transcriptomic and Proteomic Analyses
Source: Biomolecules. 2025 Aug 4;15(8):1121. doi: 10.3390/biom15081121 (PMC12383608; doi:10.3390/biom15081121)
Supplement: Supplementary file 1 [file biomolecules-15-01121-s001.zip › Suplementary Material/FileS3/PEPFOLD4/XP_065064294_11323/clusters.html]

 PEP-FOLD clusters


Download archive of all models  here   

|  |  |  |
| --- | --- | --- |
| # | file | sOPEP |
| # cluster 1 - Size: 20 |  |  |
|  | XP\_065064294\_11323-model1 | -34.4066 |
|  | XP\_065064294\_11323-model1.1 | -33.9184 |
|  | XP\_065064294\_11323-model1.2 | -33.8745 |
|  | XP\_065064294\_11323-model1.3 | -33.7031 |
|  | XP\_065064294\_11323-model1.4 | -33.572 |
|  | XP\_065064294\_11323-model1.5 | -33.5326 |
|  | XP\_065064294\_11323-model1.6 | -33.4777 |
|  | XP\_065064294\_11323-model1.7 | -33.4346 |
|  | XP\_065064294\_11323-model1.8 | -33.4288 |
|  | XP\_065064294\_11323-model1.9 | -33.3312 |
|  | XP\_065064294\_11323-model1.10 | -33.2975 |
|  | XP\_065064294\_11323-model1.11 | -33.2294 |
|  | XP\_065064294\_11323-model1.12 | -33.2263 |
|  | XP\_065064294\_11323-model1.13 | -33.1945 |
|  | XP\_065064294\_11323-model1.14 | -33.1194 |
|  | XP\_065064294\_11323-model1.15 | -33.0706 |
|  | XP\_065064294\_11323-model1.16 | -32.8943 |
|  | XP\_065064294\_11323-model1.17 | -32.784 |
|  | XP\_065064294\_11323-model1.18 | -32.7301 |
|  | XP\_065064294\_11323-model1.19 | -32.6849 |
| # cluster 2 - Size: 4 |  |  |
|  | XP\_065064294\_11323-model2 | -33.9881 |
|  | XP\_065064294\_11323-model2.1 | -33.2879 |
|  | XP\_065064294\_11323-model2.2 | -33.0522 |
|  | XP\_065064294\_11323-model2.3 | -32.809 |
| # cluster 3 - Size: 7 |  |  |
|  | XP\_065064294\_11323-model3 | -33.9435 |
|  | XP\_065064294\_11323-model3.1 | -33.8226 |
|  | XP\_065064294\_11323-model3.2 | -33.6371 |
|  | XP\_065064294\_11323-model3.3 | -33.5336 |
|  | XP\_065064294\_11323-model3.4 | -33.5131 |
|  | XP\_065064294\_11323-model3.5 | -33.1315 |
|  | XP\_065064294\_11323-model3.6 | -32.9221 |
| # cluster 4 - Size: 28 |  |  |
|  | XP\_065064294\_11323-model4 | -33.8767 |
|  | XP\_065064294\_11323-model4.1 | -33.7998 |
|  | XP\_065064294\_11323-model4.2 | -33.5095 |
|  | XP\_065064294\_11323-model4.3 | -33.4951 |
|  | XP\_065064294\_11323-model4.4 | -33.4716 |
|  | XP\_065064294\_11323-model4.5 | -33.4686 |
|  | XP\_065064294\_11323-model4.6 | -33.4642 |
|  | XP\_065064294\_11323-model4.7 | -33.4361 |
|  | XP\_065064294\_11323-model4.8 | -33.4115 |
|  | XP\_065064294\_11323-model4.9 | -33.4034 |
|  | XP\_065064294\_11323-model4.10 | -33.4021 |
|  | XP\_065064294\_11323-model4.11 | -33.3961 |
|  | XP\_065064294\_11323-model4.12 | -33.3687 |
|  | XP\_065064294\_11323-model4.13 | -33.3598 |
|  | XP\_065064294\_11323-model4.14 | -33.3498 |
|  | XP\_065064294\_11323-model4.15 | -33.3187 |
|  | XP\_065064294\_11323-model4.16 | -33.2681 |
|  | XP\_065064294\_11323-model4.17 | -33.2646 |
|  | XP\_065064294\_11323-model4.18 | -33.25 |
|  | XP\_065064294\_11323-model4.19 | -33.2445 |
|  | XP\_065064294\_11323-model4.20 | -33.2331 |
|  | XP\_065064294\_11323-model4.21 | -33.2082 |
|  | XP\_065064294\_11323-model4.22 | -33.1725 |
|  | XP\_065064294\_11323-model4.23 | -33.166 |
|  | XP\_065064294\_11323-model4.24 | -33.1341 |
|  | XP\_065064294\_11323-model4.25 | -32.8051 |
|  | XP\_065064294\_11323-model4.26 | -32.6698 |
|  | XP\_065064294\_11323-model4.27 | -32.3274 |
| # cluster 5 - Size: 9 |  |  |
|  | XP\_065064294\_11323-model5 | -33.8648 |
|  | XP\_065064294\_11323-model5.1 | -33.7522 |
|  | XP\_065064294\_11323-model5.2 | -33.6741 |
|  | XP\_065064294\_11323-model5.3 | -33.6334 |
|  | XP\_065064294\_11323-model5.4 | -33.5022 |
|  | XP\_065064294\_11323-model5.5 | -33.3448 |
|  | XP\_065064294\_11323-model5.6 | -33.2412 |
|  | XP\_065064294\_11323-model5.7 | -33.2154 |
|  | XP\_065064294\_11323-model5.8 | -33.2144 |
| # cluster 6 - Size: 9 |  |  |
|  | XP\_065064294\_11323-model6 | -33.0819 |
|  | XP\_065064294\_11323-model6.1 | -32.6751 |
|  | XP\_065064294\_11323-model6.2 | -32.4588 |
|  | XP\_065064294\_11323-model6.3 | -32.3413 |
|  | XP\_065064294\_11323-model6.4 | -32.2836 |
|  | XP\_065064294\_11323-model6.5 | -32.2355 |
|  | XP\_065064294\_11323-model6.6 | -32.0612 |
|  | XP\_065064294\_11323-model6.7 | -31.8444 |
|  | XP\_065064294\_11323-model6.8 | -31.3334 |
| # cluster 7 - Size: 6 |  |  |
|  | XP\_065064294\_11323-model7 | -33.0766 |
|  | XP\_065064294\_11323-model7.1 | -32.7696 |
|  | XP\_065064294\_11323-model7.2 | -32.5749 |
|  | XP\_065064294\_11323-model7.3 | -32.5748 |
|  | XP\_065064294\_11323-model7.4 | -32.4276 |
|  | XP\_065064294\_11323-model7.5 | -32.3663 |
| # cluster 8 - Size: 1 |  |  |
|  | XP\_065064294\_11323-model8 | -32.9055 |
| # cluster 9 - Size: 2 |  |  |
|  | XP\_065064294\_11323-model9 | -32.4365 |
|  | XP\_065064294\_11323-model9.1 | -32.2035 |
| # cluster 10 - Size: 4 |  |  |
|  | XP\_065064294\_11323-model10 | -32.3062 |
|  | XP\_065064294\_11323-model10.1 | -32.0309 |
|  | XP\_065064294\_11323-model10.2 | -31.593 |
|  | XP\_065064294\_11323-model10.3 | -31.2493 |
| # cluster 11 - Size: 6 |  |  |
|  | XP\_065064294\_11323-model11 | -32.181 |
|  | XP\_065064294\_11323-model11.1 | -32.1621 |
|  | XP\_065064294\_11323-model11.2 | -32.0141 |
|  | XP\_065064294\_11323-model11.3 | -31.9793 |
|  | XP\_065064294\_11323-model11.4 | -31.7876 |
|  | XP\_065064294\_11323-model11.5 | -31.7611 |
| # cluster 12 - Size: 1 |  |  |
|  | XP\_065064294\_11323-model12 | -31.669 |
| # cluster 13 - Size: 3 |  |  |
|  | XP\_065064294\_11323-model13 | -31.2459 |
|  | XP\_065064294\_11323-model13.1 | -30.637 |
|  | XP\_065064294\_11323-model13.2 | -29.8134 |
|  |  |  |
